# GaitCSF: Multi-Modal Gait Recognition Network Based on Channel Shuffle Regulation and Spatial-Frequency Joint Learning

**DOI:** 10.3390/s25123759

**Published:** 2025-06-16

**Authors:** Siwei Wei, Xiangyuan Xu, Dewen Liu, Chunzhi Wang, Lingyu Yan, Wangyu Wu

**Affiliations:** 1 School of Computer Science, Hubei University of Technology, Wuhan 430068, Chinaxuxiangyuan@hbut.edu.cn (X.X.);; 2 School of Management, Nanjing University of Posts and Telecommunications, Nanjing 210023, China; 3 CCCC Second Harbor Engineering Company Ltd., Wuhan 430056, China; 4 School of Computer Science, University of Liverpool, Liverpool L69 3DR, UK

**Keywords:** gait recognition, GaitCSF, computer vision, pattern recognition, multi-modal, deep learning

## Abstract

Gait recognition, as a non-contact biometric technology, offers unique advantages in scenarios requiring long-distance identification without active cooperation from subjects. However, existing gait recognition methods predominantly rely on single-modal data, which demonstrates insufficient feature expression capabilities when confronted with complex factors in real-world environments, including viewpoint variations, clothing differences, occlusion problems, and illumination changes. This paper addresses these challenges by introducing a multi-modal gait recognition network based on channel shuffle regulation and spatial-frequency joint learning, which integrates two complementary modalities (silhouette data and heatmap data) to construct a more comprehensive gait representation. The channel shuffle-based feature selective regulation module achieves cross-channel information interaction and feature enhancement through channel grouping and feature shuffling strategies. This module divides input features along the channel dimension into multiple subspaces, which undergo channel-aware and spatial-aware processing to capture dependency relationships across different dimensions. Subsequently, channel shuffling operations facilitate information exchange between different semantic groups, achieving adaptive enhancement and optimization of features with relatively low parameter overhead. The spatial-frequency joint learning module maps spatiotemporal features to the spectral domain through fast Fourier transform, effectively capturing inherent periodic patterns and long-range dependencies in gait sequences. The global receptive field advantage of frequency domain processing enables the model to transcend local spatiotemporal constraints and capture global motion patterns. Concurrently, the spatial domain processing branch balances the contributions of frequency and spatial domain information through an adaptive weighting mechanism, maintaining computational efficiency while enhancing features. Experimental results demonstrate that the proposed GaitCSF model achieves significant performance improvements on mainstream datasets including GREW, Gait3D, and SUSTech1k, breaking through the performance bottlenecks of traditional methods. The implications of this research are significant for improving the performance and robustness of gait recognition systems when implemented in practical application scenarios.

## 1. Introduction

Gait recognition [1,2,3] is a biometric technology that identifies individuals based on their walking patterns. As a long-range biometric technology in uncontrolled scenarios, it realizes identification by analyzing the dynamic patterns of human limbs while walking. Unlike other biometric methods such as face, fingerprint, and iris recognition [4,5,6], which require close-range acquisition and subject cooperation, gait recognition offers distinct advantages: it can be performed at a distance, requires no active participation from subjects, is difficult to deliberately disguise, and remains effective under varying lighting conditions and even at low resolutions. These features indicate that gait recognition has a broad application prospect in the fields of public safety monitoring, smart city construction, identity verification, access control, walking robots, etc. Especially in the scenarios that require non-intrusive long-distance monitoring, gait recognition has irreplaceable value [7,8,9,10,11].

**Figure 1:**
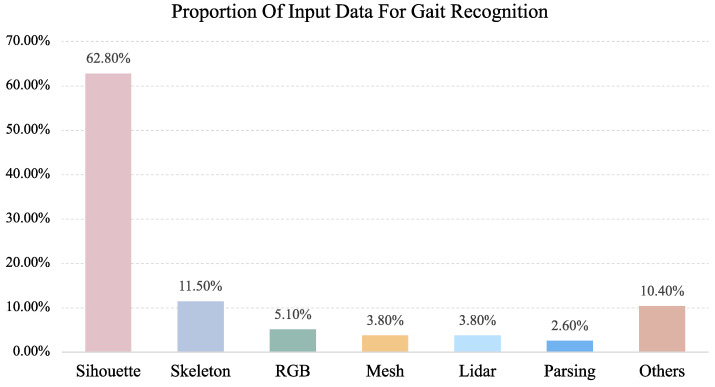
Types of different input data.

With the advancement of computer vision and deep learning technologies [12,13], gait recognition research has made significant progress in recent years. Various deep learning architectures, including convolutional neural networks (CNNs), recurrent neural networks (RNNs), and Transformers, have substantially enhanced feature extraction and representation capabilities, improving recognition accuracy in complex environments. Current gait recognition approaches can be categorized into three main streams based on their input data types, as illustrated in Figure 1: silhouette-based methods, skeleton-based methods, and multi-modal methods.

Silhouette-based gait recognition methods describe human motion characteristics from the perspective of appearance contours by extracting sequences of binarized pedestrian silhouette images. These methods simplify complex original images into binary silhouettes, effectively reducing computational complexity while mitigating interference from factors such as texture and color. Silhouette-based methods offer advantages in computational efficiency, representation conciseness, and relative simplicity of implementation. However, they are susceptible to influences from clothing changes, carried items, and viewpoint variations, potentially resulting in unstable recognition performance in complex environments. Affected by various factors, their ability to accurately reflect human motion states is significantly impacted, leading to a declining trend in recognition accuracy. Existing methods strive to extract more robust gait features from human silhouette images to mitigate the negative impact of occlusion phenomena on recognition effectiveness. Despite continuous technical innovations in these methods, the inherent limitations of silhouette data continue to constrain further improvements in recognition performance [14,15,16,17,18].

Skeleton-based methods focus on the dynamic changes of human joint points, extracting human skeleton sequences as gait features through pose estimation algorithms. By concentrating on the structural aspects of human movement rather than appearance, these methods demonstrate stronger robustness to clothing changes and minor occlusions. However, they face their own limitations: they are constrained by the accuracy of underlying pose estimation algorithms, which often introduce errors in complex backgrounds or low-resolution scenarios. Furthermore, while skeleton data effectively captures joint movement patterns, it lacks comprehensive representation of external morphological features that are crucial for distinguishing between individuals with similar movement patterns [19,20,21,22,23].

The aforementioned single-modal methods each have their advantages and disadvantages. To overcome their inherent limitations, researchers have begun to explore multi-modal fusion-based gait recognition methods. By combining the complementary advantages of silhouette features and skeleton features, multi-modal methods can construct more comprehensive and robust gait representations, maintaining stable recognition performance in complex environments. These approaches fuse skeleton and silhouette data at different levels. These multi-modal methods have, to some extent, compensated for the deficiencies of single modalities, enhancing the performance and robustness of gait recognition in complex scenarios.

Considering the inherent limitations of single-modal recognition methods, current research focus has largely shifted toward multi-modal techniques, particularly the combination of silhouette and skeleton information. The core value lies in the complementary features between these two modalities: silhouette data captures complete information about human external shape and dynamic changes, while skeleton data provides stable representations of key structural points of the human body. By integrating these two essentially different but complementary information sources, fusion models can construct more comprehensive and interference-resistant gait feature representations, effectively breaking through the performance ceiling of single-modal data. Recent research findings [24,25,26,27,28] confirm that this multi-modal fusion framework has opened up a highly promising technical pathway for the field of gait recognition, driving both theoretical innovation and practical application in this domain.

However, existing multi-modal methods still have deficiencies in feature fusion and modal complementarity: on one hand, they lack effective modeling of the periodic features in gait sequences, making it difficult to comprehensively capture temporal dynamic information in gait; on the other hand, inter-modal information interaction is insufficient, often remaining at the level of feature concatenation or simple weighted fusion, which hampers deep-level feature fusion and complementary enhancement; furthermore, they have limited capability to capture long-range dependencies in complex environments, particularly performing poorly when processing longer sequences or irregular gait patterns; finally, existing methods predominantly focus on spatial domain feature extraction, neglecting the potential value of frequency domain information for representing periodic gait patterns.

**Figure 2:**
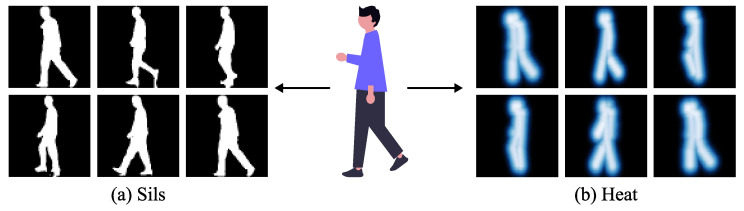
Silhouette and pose heatmap.

Based on the above experimental observations, we have developed a novel multi-modal gait recognition framework (GaitCSF) that combines channel shuffling regulation technology with spatial-frequency joint learning strategies, aimed at generating robust and comprehensive gait feature representations. Our method adopts a dual-modal feature representation approach using silhouettes and skeleton heatmaps, as shown in Figure 2, utilizing original silhouette data to preserve complete pedestrian shape features, while transforming skeleton information into heatmap form to enhance the expression of key structural points. The advantage of this dual-modal architecture lies in the fact that silhouettes can completely preserve the overall human body shape and movement changes, while skeleton heatmaps reflect the frequency and intensity of activities in various body parts through heat distributions of different intensities, allowing skeleton information to be expressed in a richer form. This unique feature fusion method both avoids the limitations of single modalities and enhances the expressive depth of skeleton features through heatmap technology, thereby providing a better solution for gait recognition tasks.

To address these issues, this paper proposes a multi-modal gait recognition network based on channel shuffling regulation and spatial-frequency joint learning (GaitCSF), which achieves effective fusion and complementary enhancement of silhouette features and heatmap features through innovative frequency-domain transformation feature enhancement and shuffling channel selective regulation, combined with a multi-branch parallel feature extraction architecture. The main contributions of this paper can be summarized in four aspects:

The main contributions of this paper are as follows:

- We employed a three-branch parallel feature extraction architecture to optimize contour features, heatmap features, and their fusion features, thereby achieving complementary enhancement and effective fusion of multi-modal information. Compared to simple early or late fusion strategies, this divide-and-conquer parallel processing method allows each modality’s features to first fully develop within their respective feature spaces, preserving modality-specific discriminative information, and then fuse at a high semantic level, thereby maximizing the advantages of modality complementarity. This design fully considers the hierarchical and multi-scale characteristics of gait features, enabling the capture of multi-level gait information from local details to global structures, thereby providing a more comprehensive and rich feature representation.
- We design a channel shuffle-based feature selective regulation module that achieves adaptive feature enhancement under lightweight parameter design through channel grouping and feature shuffling strategies. This module innovatively divides input features along the channel dimension into multiple subspaces, which undergo channel-aware and spatial-aware processing, respectively, to capture dependencies across different dimensions, and then promotes information exchange between different semantic groups through channel shuffling operations. This design not only reduces computational complexity but also enhances the discriminability of feature representations, making it particularly suitable for processing the fusion and optimization of multi-modal features. Compared to traditional feature enhancement methods, CFS is more efficient and has stronger expressive capability, achieving better feature optimization effects with limited computational resources. Especially when processing multi-modal data in gait recognition, this lightweight yet effective feature regulation mechanism can highlight key gait features, suppress interference information, and improve the model’s recognition accuracy and robustness.
- We propose a spatial-frequency joint learning module (SFL) that effectively captures periodic patterns and global dependencies in sequences by mapping gait features to the frequency domain through Fourier transform. Frequency domain processing has the natural advantage of a global receptive field, capable of transcending the spatial limitations of local convolution operations to capture overall movement patterns and periodic features in gait. SFL designs a dual-pathway architecture, with one pathway processing features in the spatial domain and the other processing frequency domain information through FFT transformation, ultimately integrating the advantages of both through an adaptive fusion mechanism to enhance the model’s ability to express gait temporal patterns. SFL can not only process traditional periodic gait features, it can also effectively address irregular gait patterns, such as changes in walking speed, turning, pausing, and other complex behaviors common in real-world scenarios, improving the model’s adaptability in practical applications.
- Comprehensive experimental evaluations are conducted on three mainstream datasets (GREW [29], Gait3D [30], and SUSTech1k [31]) and the results show that the proposed GaitCSF model outperforms existing methods across all metrics, demonstrating stronger robustness and recognition accuracy, especially under complex environmental conditions. In addition, through systematic ablation experiments, we verify the effectiveness and contribution of each module, providing valuable references for the design and optimization of multi-modal gait recognition methods.

This paper is structured as follows: In Section 2, we review the literature related to gait recognition, encompassing silhouette-based approaches, skeleton-based techniques, and multi-modal methodologies. Section 3 details the proposed GaitCSF model architecture, including the channel shuffle-based feature selective regulation module and the spatial-frequency joint learning module. Section 4 presents experimental results and analysis, including comparisons with existing methods and ablation experiments. Finally, Section 5 summarizes the paper and points out directions for future research.

## 2. Related Work

This section will briefly outline the development trajectory and technical categories of gait recognition models. After experiencing multiple stages of evolution, the mainstream technical approaches in this field can be categorized into three types: silhouette-based methods, skeleton-based methods, and multi-modal methods.

### 2.1. Silhouette Based Gait Recognition

Silhouette-based gait recognition methods extract human contour features as initial input, learning gait features from silhouettes to obtain rich gait characteristics. GaitSet [14] models gait as an unordered set of frames, breaking through the limitations of traditional template-based or sequence-based methods. By constructing a global–local fusion deep network and utilizing convolutional neural networks (CNNs) to extract frame-level local features, this method demonstrates significant robustness under cross-view, occlusion, and complex walking conditions. GaitPart [15] divides human walking motion into distinct local segments, enabling independent analysis and modeling of each component, breaking through the limitations of traditional methods that use the whole silhouette as a unit, and proposes a spatiotemporal feature learning framework based on short-term micro-actions. This method innovatively designs a focal convolution layer, enhancing fine-grained spatial feature extraction for various body parts (such as arm swinging, leg posture) through layered constraints on the receptive field; meanwhile, it develops a micro-action capture module (MCM), employing multi-scale sliding windows to aggregate local temporal dynamics, combined with a channel attention mechanism to strengthen key motion patterns. GaitGL [18] innovatively proposes a global–local dual-branch convolution layer (GLCL), resolving the limitations of traditional methods that solely rely on either global or local representations by extracting global contextual relationships and local detailed features in parallel. This framework introduces a mask-driven local feature learning mechanism, designing multi-level (part, strip, pixel) complementary masks to randomly occlude feature maps, forcing the network to fully explore dynamic details from different regions during training, breaking through the information bottleneck of fixed partitioning strategies. Simultaneously, it proposes a local temporal aggregation module (LTA), compressing redundant frame information in the temporal dimension while preserving high-resolution spatial features, enhancing the spatiotemporal modeling capability of gait sequences. QAGait [32] reconstructs the gait recognition process from a quality assessment perspective, offering systematic solutions for low-quality silhouettes (such as abnormal segmentation, severe occlusion) and high-quality samples with posture deviations in real-world scenarios. This framework innovatively designs a three-stage quality filtering strategy: maximal connect area analysis to remove background noise, template match based on Hu moments to eliminate non-human-shaped contours, and lean-aware alignment to correct non-standard postures. It also introduces quality-aware loss functions QACE and QATriplet, dynamically adjusting classification boundaries and triplet margins using local feature norms, achieving quality-adaptive optimization in feature space. GEI [16] pioneered the Gait Energy Image method, which innovatively compresses temporal silhouette information into two-dimensional space, generating compact gait representations through temporal dimension normalization. This representation not only effectively suppresses single-frame silhouette noise but also significantly reduces computational complexity, providing a feasible solution for real-time gait recognition in video surveillance scenarios. GaitEdge [17] constructs an end-to-end synthesis-recognition joint framework, generating background-independent standardized silhouettes through a differentiable gait alignment module, effectively eliminating the interference of RGB domain noise on feature extraction. Although silhouette-based methods have made significant progress, silhouette data is susceptible to clothing occlusion and complex environments, leading to losses during feature extraction, which remain key bottlenecks constraining the performance improvement of silhouette-based gait recognition methods.

### 2.2. Skeleton-Based Gait Recognition

Skeleton-based gait recognition methods focus on human structure, primarily extracting features by analyzing the dynamic movement patterns of various body parts, and commonly using human key points and skeletal connection graphs as basic input features. PoseMapGait [20] employs pose estimation maps as feature input sources, fusing heatmaps with pose temporal evolution features. By predicting pose estimation maps for various human body regions, coupled with graph convolutional network techniques to capture spatiotemporal information, it significantly improves the stability of gait recognition in complex environments, maintaining good performance even in cases of significant subject clothing changes and background interference. PoseGait [19] constructs gait representations based on 3D human pose information, combining human prior knowledge to design an anti-interference gait recognition framework. This method demonstrates strong adaptability when facing viewpoint diversity and clothing changes, while maintaining considerable recognition accuracy even with lower feature dimensions. GaitGraph [21] explores the application of graph convolutional networks in gait recognition, effectively capturing topological relationships between human joint points through graphical modeling of skeletal structures, enhancing the expressiveness of gait features and showing unique advantages in dynamic behavior analysis. DyGait [23] emphasizes information mining in the temporal dimension, focusing on dynamic modeling of joint motion trajectories, enhancing the temporal expressive capability of gait features through precise capture of trajectory information, and providing richer feature descriptions in dynamically complex scenarios. GPGait [33] achieves unified representation of pose sequences through human-oriented transformation (HOT), generates multi-feature expressions of joints, bones, and angles through human-oriented descriptors (HOD), and extracts local–global relationships through part-aware graph convolutional networks (PAGCNs). SkeletonGait [22] proposes innovative skeleton mapping representation techniques, employing Gaussian approximation algorithms to convert joint coordinates into heatmap form, demonstrating stronger anti-interference capabilities compared to traditional coordinate representations, and maintaining high recognition performance even under conditions of occlusion and unstable lighting.

Although skeleton-based methods have obvious advantages in addressing viewpoint changes and appearance differences, it cannot be ignored that, compared to silhouette images, skeletal data has certain limitations in carrying body morphological information, especially in low-resolution scenarios where joint localization errors can lead to feature degradation, becoming a key bottleneck constraining their practical application.

### 2.3. Multi-Modal Based Gait Recognition

Traditional gait recognition methods primarily rely on single-modal data, but all have obvious limitations. Silhouette-based methods, while capable of capturing overall body shape and dynamic changes, perform poorly in complex backgrounds, lighting variations, and occlusion situations, and are extremely sensitive to clothing changes, making it difficult to extract stable identity features. Skeleton-based methods, despite having certain robustness under different clothing and viewpoints, lack sufficient body shape information in skeletal data, and keypoint detection is prone to errors in complex scenarios, leading to inaccurate skeleton feature extraction.

In response to the limitations of single modalities, multi-modal gait recognition methods have emerged, comprehensively capturing human gait features by fusing the complementary advantages of different data sources. HybridGait [24] focuses on cross-clothing variation scenarios, creating the CCGait benchmark dataset and proposing a hybrid framework that combines 3D human mesh with 2D information through temporal dynamic capture and spatial projection techniques, effectively addressing challenges brought by clothing and viewpoint variations. Experimental results indicate that this method significantly outperforms single-modal methods under cross-clothing conditions. GaitCode [25] explores the fusion potential of wearable sensor data, combining accelerometer and ground contact force data. Through autoencoders for early or late sensor fusion, this method greatly enhances gait feature expression capability, achieving extremely low error rates across multiple evaluation metrics. This achievement demonstrates the effectiveness of multi-modal data fusion in improving gait recognition accuracy, especially in scenarios where traditional visual methods are difficult to apply. The MSAFF [26] framework innovatively proposes a multi-stage feature fusion strategy, capturing fine-grained gait information through a multi-scale spatial–temporal feature extraction (MSSTFE) module, and enhancing semantic associations between skeleton and silhouette data through an adaptive feature fusion module (AFFM). This design fully utilizes the complementarity of different modal data in spatial and temporal dimensions. GaitRef [34] proposes a new gait recognition method combining silhouette and skeleton data. Addressing the limitations of traditional methods—silhouettes being affected by clothing and carried items, while skeleton detection suffers from inter-frame jitter issues—this research designs two models: GaitMix and GaitRef. GaitMix directly fuses features from both modalities, while GaitRef further utilizes the temporal consistency of silhouette sequences to optimize skeleton quality. GaitSTR [35] proposes a dual-stream rectification method for gait recognition. By combining skeletal and silhouette modalities, it uses self-rectification and cross-modal rectification to improve skeletal representation quality. GaitSTR decomposes skeletons into joint points and bone connection representations, performing internal fusion in a graph convolutional network, while utilizing the temporal consistency of silhouette sequences to guide skeletal rectification, effectively reducing inconsistencies in skeleton detection and improving gait recognition accuracy. Evolutionary algorithm-based Fusion [28] explores more complex fusion strategies, using 3D-CNN to process video data, LSTM to process inertial sensor data, and intelligent decision fusion through the Grey Wolf Optimization algorithm (GWO). This approach not only improves recognition accuracy but also enhances system adaptability in dynamic environments. MMGaitFormer [27], based on the Transformer architecture, designs specialized spatial fusion modules (SFMs) and temporal fusion modules (TFMs) to fuse skeleton and silhouette data at different levels. This method effectively handles long sequence dependencies through attention mechanisms while preserving the unique advantages of multi-modal data.

These multi-modal gait recognition methods effectively overcome the limitations of single modalities by fusing the advantages of different data sources, providing new ideas for high-precision human identification in complex scenarios and demonstrating broad application prospects.

## 3. GaitCSF

In this section, we will provide an in-depth introduction to our proposed gait recognition network GaitCSF, with emphasis on its core modules: the channel shuffle-based feature selective regulation module (CFS) and the spatial-frequency joint learning module (SFL).

### 3.1. Pipeline

**Figure 3:**
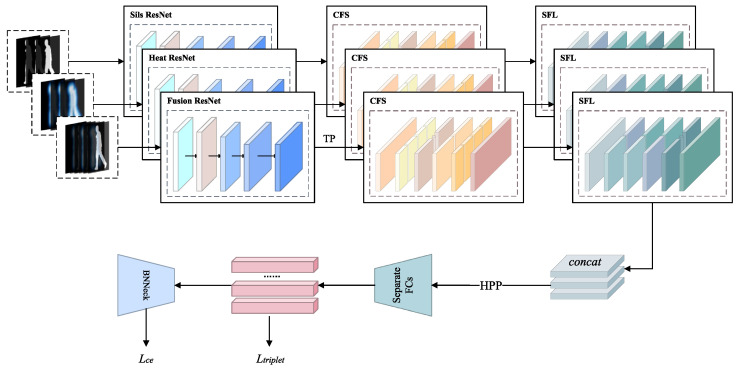
Overall flow of GaitCSF.

Figure 3 illustrates our proposed gait recognition network. As shown in Figure 3, GaitCSF first receives dual-modal inputs of silhouette images and heatmaps, reconstructing them into standard five-dimensional tensors: [N,C,S,H,W].

The model deploys three independent ResNet backbone networks to process silhouette features, heatmap features, and their cross-modal fusion features, where fusion features achieve multi-source information complementarity through channel dimension concatenation. Subsequently, temporal pooling (TP) is employed to aggregate global features along the sequence dimension, generating four-dimensional tensors with global representation capability: [N,C,H,W].

The CFS then enhances feature expression through fine-tuning of features from each branch via a group processing mechanism. This module first divides features into multiple subgroups along the channel dimension, with each subgroup further divided into two parallel branches. One branch extracts channel statistical information through global average pooling, capturing inter-channel dependencies, and scales and shifts the generated features using parameters; the other branch utilizes group normalization to generate spatial statistical information, performing similar feature enhancement. The two branches are dynamically adjusted through learnable parameters, producing more discriminative feature representations. Finally, the module applies a “channel shuffling” operation to enable communication between different semantic information, achieving global context-aware feature enhancement, which significantly improves feature discriminability while introducing only a minimal number of parameters.

Subsequently, the model employs SFL to further enhance feature representation capability. This module constructs a bidirectional processing architecture for spatial and frequency domains, extracting complementary information from both spatial and frequency domains. The frequency pathway maps features to the spectral space through Fourier transform, leveraging the global receptive field advantage of frequency domain processing to capture global periodic patterns; the spatial pathway employs multi-layer convolutional networks to extract local detail features. For inputs with different periodicities, SFL adaptively balances the contribution weights of the two pathways through spectral energy distribution analysis. The internal spectral attention mechanism of the module can selectively enhance discriminative frequency components while suppressing high-frequency noise interference, ultimately integrating spatial and frequency domain information through learnable fusion strategies to generate robust feature representations.

Multi-branch features, after channel concatenation, are input to horizontal pyramid pooling (HPP) for processing, segmenting features into feature vectors of multiple local regions. Finally, the model generates metric learning embedding vectors and classification logits through fully connected layers (FC) and batch normalization neck networks (BNNeck), jointly optimizing triplet loss and cross-entropy loss. The entire process can be expressed by the following formula:

(1) $$\begin{array}{cc} E_{out}= & SFL(CFS(TP(N(e)))) \end{array}$$

where *e* represents silhouette, heatmaps, and fusion feature maps; N(·) represents ResNet-Like feature extraction operations; TP(·) represents temporal pooling operations; CFS(·) represents feature selective regulation operations; SFL(·) represents spatial-frequency joint learning operations; $X_{out}$ represents the output silhouette features, heat features, and fusion features. Here,

(2) $$\begin{array}{c} fusion=sils⊕heat\; \end{array}$$

(3) $$\begin{array}{c} X_{out}=\mathrm{BNN}\left(\mathrm{FC}\left(\mathrm{HPP}\left(connect\right)\right)\right)\;\; \end{array}$$

where $connect=sils_{out}⊕heat_{out}⊕fusion_{out}$ represents the aggregated features; HPP(·) represents horizontal pyramid pooling operations; FC(·) represents fully connected layers; BNN(·) represents normalization operations.

### 3.2. ResNet-like

**Figure 4:**
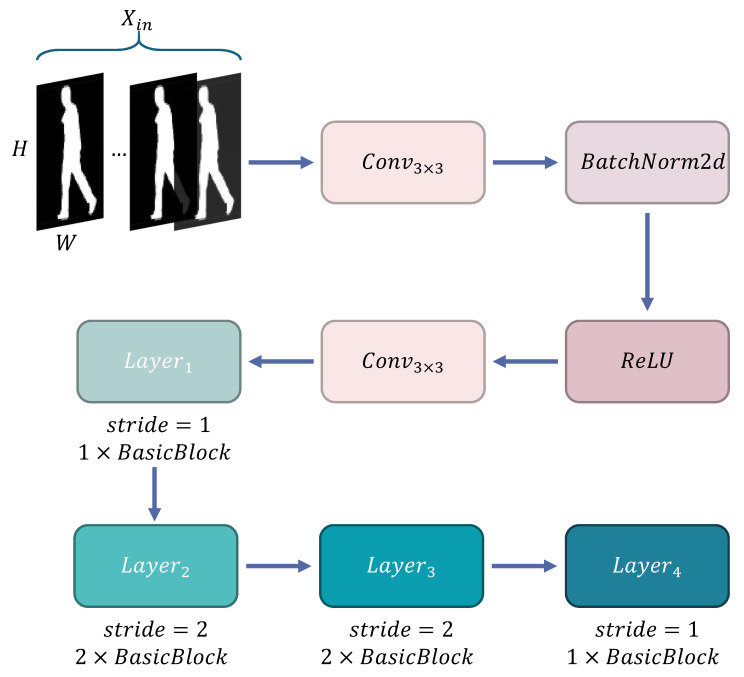
Feature extraction backbone network ResNet-Like.

**Figure 5:**
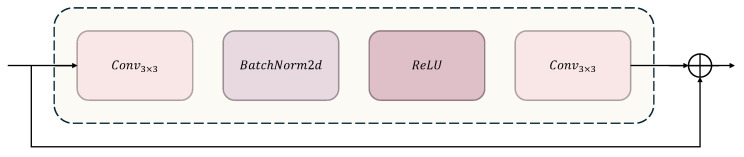
A BasicBlock.

With the iterative evolution of convolutional neural network technology, breakthrough designs of classic architectures continue to drive the development of visual tasks. CNN architectures have undergone an evolution process from simple to complex, with representative ones being AlexNet [36], VGG-16 [37], and ResNet [38]. In the field of gait recognition, the shallow convolutional stacking structures [39,40] used by early researchers had limitations in feature expression capability. In GaitCSF, we borrowed the ResNet-like backbone proposed by GaitBase [41] as our model’s feature extractor, constructing a backbone network with hierarchical feature extraction capability, as shown in Figure 4. This network takes feature maps of size 64 × 44 as input and designs a feature initialization module for spatial mapping. Input features first go through a mapping layer consisting of 3 × 3 convolution kernels (stride = 1, padding = 1), extending the channel dimension to 32 dimensions, followed by batch normalization and ReLU activation function for non-linear transformation, outputting primary feature representation with dimensions (N, 32, 64, 44). The main body of the network adopts a four-stage progressive architecture, with each stage implementing progressive enhancement of feature hierarchy through several basic residual units (BasicBlock), as shown in Figure 5. Layer1 extracts local features through a single BasicBlock while maintaining 64 × 44 spatial resolution. Layer2 and Layer3 each have two BasicBlocks, utilizing stride = 2 convolution to achieve feature map downsampling, synchronously doubling channel dimensions to 64 and 128 dimensions, with feature map sizes compressed to 32 × 22 and 16 × 11, respectively. Layer4 adopts a single BasicBlock to increase channel dimension to 256 dimensions, outputting high-order feature tensor (N, 256, 16, 11). This hierarchical progressive design strategy enhances model representation capability by balancing decreasing spatial resolution and increasing channel numbers while maintaining reasonable computational overhead. The multi-scale architecture combined with residual connections captures both microscopic details and macroscopic semantic information of gait sequences, providing rich feature representation for downstream recognition tasks. Mathematically, this entire process can be represented as follows:

(4) $$\begin{array}{c} e_{0}=\mathrm{ReLU}\left(\mathrm{BN}\left(s_{0}\cdot e+a_{0}\right)\right) \end{array}$$

(5) $$\begin{array}{c} E=\mathrm{ReLU}\left\{F\left(e,\{s_{i}\}\right)+e\right\} \end{array}$$

(6) $$\begin{array}{c} E=\mathrm{Layer}4(\mathrm{Layer}3\left(\mathrm{Layer}2\left(\mathrm{Layer}1\left(e_{0}\right)\right)\right)) \end{array}$$

### 3.3. Channel Shuffle-Based Feature Selective Regulation Module

**Figure 6:**
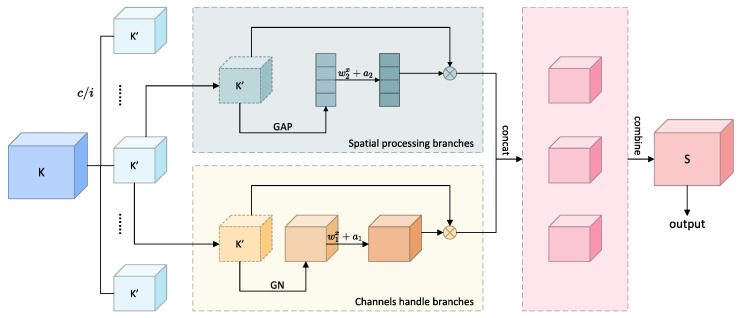
Schematic diagram of the channel shuffle-based feature selective regulation module.

In gait recognition research, effectively extracting and integrating multi-modal feature information faces two key challenges: on one hand, it needs to simultaneously capture dependencies in spatial and channel dimensions to obtain comprehensive gait feature expression; on the other hand, it needs to maintain model lightweight design to adapt to practical application scenarios. Traditional methods often have computational redundancy or insufficient feature expression capability when processing such spatial-channel correlations. The fundamental challenge lies in the inherent semantic heterogeneity between silhouette and heatmap modalities, where simply concatenating or averaging features often leads to suboptimal performance as important modal-specific characteristics may be diluted or lost. Moreover, different feature channels carry varying levels of discriminative information, but existing approaches typically treat all channels equally, failing to emphasize crucial gait-specific patterns. Addressing these challenges, we propose a feature selective regulation module based on channel shuffling, which achieves fine-tuning of different modal features through combining feature grouping strategies and channel shuffling mechanisms with extremely low parameter overhead. CFS divides input features along the channel dimension into multiple subspaces, each processed through a dual-path processing architecture—one path capturing inter-channel correlations, the other focusing on spatial context information—finally promoting information exchange between different semantic subspaces through channel shuffling [42] operations, achieving efficient enhancement of cross-modal features, as shown in Figure 6.

The theoretical foundation of CFS is as follows:

Given input feature $K\in P^{N\times C\times H\times W}$, the CFS module first divides its channel dimension into *i* groups of subspaces, obtaining $K=\left[K_{1},\ldots ,K_{i}\right],K_{x}\in P^{N\times (C/i)\times H\times W}$. Each subspace $K_{x}$ undergoes dual parallel processing. In CFS, the spatial statistics information branch utilizes group normalization and learnable parameters for dynamic capture of local spatial associations. Through using parametric scaling factor $W_{1}^{x}$ and bias term $a_{1}^{x}$ to process normalized features, its mathematical expression is as follows:

(7) $$\begin{array}{c} K_{x}^{sp}=\mathrm{GN}(K_{x})⊙\sigma \left(W_{1}^{x}\cdot GN\left(K_{x}\right)+a_{1}^{x}\right) \end{array}$$

where GN represents group normalization operation, ⊙ represents the channel-wise product, and $W_{1}^{x}\in P^{(C/2i)\times 1}$ is the spatial sensitivity parameter.

The inter-channel correlation branch implements global statistics and dynamic scaling. By compressing spatial information through global average pooling (GAP) on features of each subspace, generating channel-level statistics $Q_{x}\in P^{C/2i}$, then generating channel weight vectors through fully connected layers and Sigmoid function, its mathematical expression is

(8) $$\begin{array}{c} Q_{x}=\sigma \left(W_{2}^{x}\cdot \mathrm{GAP}(K_{x})+a_{2}^{x}\right) \end{array}$$

(9) $$\begin{array}{c} \;K_{x}^{ch}=X_{k}⊙Expand\left(Q_{x}\right) \end{array}$$

where GAP is global average pooling, $W_{2}^{x}\in P^{(C/2i)\times 1}$ and $a_{2}^{x}$ are learnable parameters, σ is the Sigmoid function, and Expand(·) expands scalar weight vectors to the same dimensions as the original feature maps.

Dual-path outputs undergo matrix transposition operations after channel concatenation:

(10) $$\begin{array}{c} K_{x}^{{\,}^{\prime }}=Concat\left(K_{x}^{ch},K_{x}^{sp}\right)\cdot F \end{array}$$

where the transposition matrix $F\in {\left\{0,1\right\}}^{\left(C/i)\times (C/i\right)}$ satisfies $\frac{\partial L}{\partial F}\neq 0$, ensuring that the shuffling operation maintains learnability in backpropagation. The final output preserves the original feature distribution through residual connections:

(11) $$\begin{array}{c} K_{out}=K+\sum_{x=1}^{i}K_{x}^{{\,}^{\prime }} \end{array}$$

All enhanced sub-features are aggregated, and cross-group information exchange is implemented through channel shuffling operations: $Y=Channel\_Shuffle(\left[K1^{{\,}^{\prime }},K2^{{\,}^{\prime }},\ldots ,Ki^{{\,}^{\prime }}\right])$, where channel shuffling operations rearrange feature channels so that each output channel contains information from different subgroups, significantly enhancing the discriminative power of features.

### 3.4. Spatial-Frequency Joint Learning Module

**Figure 7:**
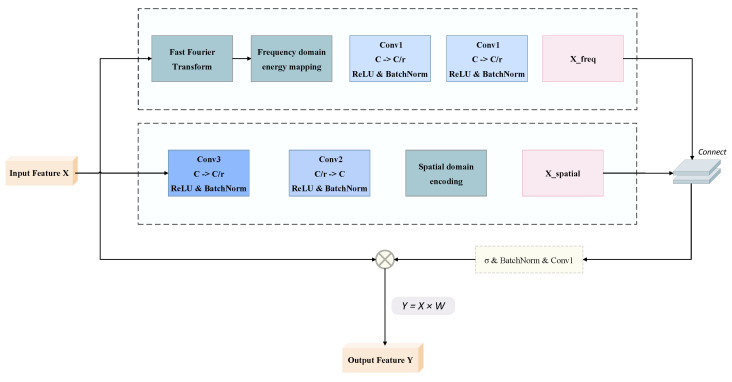
Spatial-frequency joint learning module.

Gait recognition research often faces the problem of incomplete feature expression, especially limitations in capturing periodic patterns and global dependencies in time series, while the effective fusion of global and local features is key to improving recognition accuracy. Traditional spatial domain feature extraction methods, while effective at capturing local detail information, struggle to model correlations between distant pixels, which are essential for complete expression of gait features. This limitation is particularly critical for gait recognition because human walking inherently exhibits strong periodicity and long-range temporal dependencies that spatial-only processing cannot adequately capture. Additionally, existing approaches predominantly operate in the spatial domain, overlooking the rich discriminative information contained in the frequency domain that could effectively characterize the rhythmic and cyclical nature of human gait patterns across different individuals. Our proposed spatial-frequency joint learning module addresses this issue by simultaneously processing information in frequency and spatial domains. This module effectively integrates information from different modalities through frequency domain transformation and spatial processing branches, thereby enhancing gait feature representation. SFL is particularly suitable for processing gait data because gait sequences inherently have obvious periodicity and global correlation, and frequency domain analysis can effectively capture this feature and can capture long-range dependencies and periodic patterns in gait sequences. As shown in Figure 7, this module first constructs a dual-path architecture, with one path processing features in the spatial domain and the other mapping features to the spectral space through fast Fourier transform, then fusing complementary information from both domains through an adaptive weighting mechanism, ultimately generating features with higher discriminative power.

The theoretical foundation of SFL is as follows:

Given input feature $X\in R^{N\times C\times H\times W}$ projected to the frequency domain processing branch through fast Fourier transform (FFT), decomposing spatial domain signals into frequency components:

(12) $$\begin{array}{c} X_{freq}=Ffft\left(X\right)=|FFT2D\left(X\right)| \end{array}$$

where FFT2D represents fast Fourier transform, and |·| represents complex magnitude calculation. Frequency domain features are subsequently enhanced through parametric transformation networks:

(13) $$\begin{array}{c} X_{\left\{freq\_\{enhanced\}\right\}}=\Phi_{freq}\left(X_{\left\{freq\right\}}\right) \end{array}$$

where transformation function $\Phi_{freq}$ consists of structured convolutional layers:

(14) $$\begin{array}{c} \Phi_{freq}\left(X\right)=ReLU\left(BN\left(Conv2\left(ReLU\left(BN\left(Conv1\left(X\right)\right)\right)\right)\right)\right) \end{array}$$

Conv1 reduces channel dimension from *C* to C/r (*r* is the reduction ratio), and Conv2 restores to the original dimension, effectively controlling computational complexity.

Meanwhile, the spatial processing branch transforms the original features:

(15) $$\begin{array}{c} X_{spatial}=\Phi_{spatial}\left(X\right) \end{array}$$

The spatial transformation network $\Phi_{spatial}$ adopts an expanded receptive field design:

(16) $$\begin{array}{c} \Phi_{spatial}\left(X\right)=ReLU\left(BN\left(Conv1\times 1\left(ReLU\left(BN\left(Conv3\times 3\left(X\right)\right)\right)\right)\right)\right) \end{array}$$

Features from both branches are integrated into a unified representation through a fusion function. The dimension of the fused features becomes 2C, containing complementary information from frequency and spatial domains. Subsequently, feature modulation coefficients are generated through the transformation network Ψ:

(17) $$\begin{array}{c} X_{\left\{fusion\right\}}=Concat\left(X_{\left\{freq\_enhanced\right\}},X_{\left\{spatial\right\}}\right) \end{array}$$

(18) $$\begin{array}{c} W=\sigma \left(\Psi \left(X_{\left\{fusion\right\}}\right)\right)=\sigma \left(BN\left(Conv\left(X_{\left\{fusion\right\}}\right)\right)\right) \end{array}$$

where σ is the Sigmoid activation function, mapping output to the (0,1) interval. The final output is obtained through adaptive modulation:

(19) $$\begin{array}{c} Y=W\cdot X \end{array}$$

## 4. Experiments

In this section, we conduct a thorough performance assessment of the GaitCSF model across three leading datasets: Grew [29], Gait3D [30], and SUSTech1k [31]. First, we detail the characteristics of these datasets and their corresponding training configurations. Subsequently, we compare and analyze GaitCSF with state-of-the-art gait recognition methods under the same experimental conditions. Finally, through a series of ablation experiments, we systematically analyze the specific contributions and impacts of each component of GaitCSF on the overall model performance.

### 4.1. Datasets

Our experiments are based on three representative datasets, covering various application scenarios from complex outdoor environments to controlled laboratory conditions. We adopted the Grew and Gait3D datasets applicable to large-scale outdoor environments, as well as the small dataset SUSTech1k from Southern University of Science and Technology for evaluation. To illustrate the scale and complexity of each dataset, Table 1 displays key statistical metrics, including identity count and total sequence numbers.

The Grew is a large-scale gait library built based on natural videos, covering thousands of hours of video streams captured by hundreds of cameras. This dataset is massive, containing 26,345 independent identity labels and 128,671 gait sequences, and provides four modalities of data: human silhouette images, optical flow information, 2D and 3D human key points. Structurally, Grew is divided into four subsets: training set (20,000 identities, 102,887 sequences), validation set (345 identities, 1784 sequences), test set (6000 identities, 24,000 sequences), and interference set. The evaluation protocol stipulates that each test identity is assigned 4 sequences, with 2 as query samples and the other 2 constituting the gallery set. Additionally, 233,857 extra gait sequences are introduced as interference samples to simulate the recognition difficulty in large-scale practical applications.

The Gait3D is specifically designed for 3D gait recognition research, aiming to provide dense 3D human representation data. The collection environment is set in a real supermarket scene, recording 25,309 gait sequences of 4000 different identities through 39 distributed cameras. This dataset provides rich multi-modal information, including 2D representations, 3D representations, and original RGB video frames. In the experimental division, Gait3D uses 3000 identities (18,940 sequences) for model training, with the remaining 1000 identities (6369 sequences) for performance evaluation. During testing, one sequence is randomly extracted from each identity as a query sample, with all remaining sequences of the same identity constituting the retrieval library. This dataset particularly focuses on capturing complex factors in real-world scenarios, including occlusion phenomena, dynamic background environments, multi-viewpoint changes, and irregular gait patterns, providing an ideal platform for evaluating model robustness in practical application environments.

As the pioneering large-scale LiDAR-based gait recognition dataset, SUSTech1K demonstrates the viability of using point cloud data for biometric identification. It encompasses 25,239 gait sequences from 1050 distinct individuals, offering three essential data formats: high-resolution 3D point clouds captured via LiDAR, RGB images (complete with silhouette annotations), and synchronized frame data. The dataset adopts a tripartite strategy: 250 identities (6011 sequences) for training, another 250 identities (6025 sequences) constituting the validation set, and the remaining 550 identities (13,203 sequences) as the test set. The evaluation scheme specifically designed attribute-based subdivided test sets, including changes in carried items, clothing differences, and occlusion levels, aiming to comprehensively evaluate the model’s ability to handle various practical interference factors.

In our evaluation process, we strictly implement the original protocols established upon each dataset’s release, following official standards for training, testing, and dataset partitioning. To assess recognition performance, we primarily employ Rank-k accuracy metrics (k∈1,5,10,20).

### 4.2. Implementation Details

Table 2 displays the hyperparameter configurations employed for GaitCSF experiments on various datasets. These settings will serve as our training parameters.

#### 4.2.1. Training Batch Size

In experiments, the training batch is (N, S), where N represents the number of different identity labels contained in each batch, and S represents the number of sequence samples corresponding to each identity. For large-scale Grew and Gait3D, we set the batch size to (32, 4), i.e., each batch contains 32 different identities, with 2 sequence samples selected for each identity. For the relatively smaller SUSTech1K dataset, the batch configuration is adjusted to (8, 16), i.e., each batch contains 8 different identities, but 16 sequence samples are selected for each identity for training.

#### 4.2.2. Optimizer

For parameter optimization during model training, we implement stochastic gradient descent (SGD) with 0.05 as the starting learning rate, 0.9 as the momentum coefficient, and 0.0005 as the weight decay parameter. For different datasets, we design specific learning rate scheduling strategies. For the Grew, we implement 180K iterations of training, reducing the learning rate to one-tenth of its original value at the 80 K, 120 K, and 150 K iterations; the Gait3D training totals 60,000 iterations, with the learning rate decreasing to one-tenth of its current value sequentially at the 20 K, 40 K, and 50 K iterations; for the SUSTech1K, we execute 50 K iterations of training, applying the same learning rate decay strategy at the 20 K and 40 K iterations.

#### 4.2.3. Other

To address the visual noise issue in the Grew, we adopt probabilistic data augmentation techniques. Each input sample has a 20% probability of receiving random perspective transformation, horizontal flipping, rotation processing, or region erasure operations, respectively. This augmentation mechanism effectively improves the model’s adaptability to visual changes, significantly enhancing the model’s stability and generalization capability in complex real-world environments.

Our experimental setup utilized the PyTorch deep learning framework (version 1.13.1) with CUDA 11.7, deployed across 8 NVIDIA GeForce RTX 1070TI GPUs (64 G memory) (NVIDIA, Santa Clara, CA, USA) for this research implementation.

### 4.3. Comparison with Other Methods

To comprehensively evaluate the performance of the proposed GaitCSF model, this section will conduct comparative experiments on three representative datasets: the large-scale outdoor dataset GREW, the real supermarket scene dataset Gait3D, and the multi-attribute test dataset SUSTech1k. These datasets cover various application scenarios from complex outdoor environments to controlled laboratory conditions, capable of comprehensively verifying the model’s recognition ability under different challenging conditions. We will compare and analyze GaitCSF with current leading gait recognition methods, including silhouette-based methods, skeleton-based methods, and multi-modal methods, to verify the effectiveness and advancement of the model proposed in this paper.

#### 4.3.1. Evaluate on Grew Dataset

Experimental results show that GaitCSF significantly outperforms all comparison methods on the GREW, as shown in Table 3. Compared to the silhouette-based GaitRGA, GaitCSF’s Rank-1 recognition rate improved by 7.58%; compared to the skeleton-based method GPGait, it improved by 21.86%; compared to the multi-modal method GaitSTR, it improved by 11.46%. This significant performance advantage fully validates the effectiveness of the network proposed in this paper. CFS achieves fine-tuning and adaptive enhancement of multi-modal features through channel grouping and feature shuffling strategies. SFL effectively captures the periodic patterns and global dependencies in gait sequences in the GREW by mapping gait features to the frequency domain through Fourier transform. Overall, GaitCSF’s excellent performance on the GREW validates its capability in handling large-scale outdoor complex scene gait recognition tasks, especially its robustness and accuracy in addressing practical challenges such as viewpoint changes, background interference, and lighting changes.

#### 4.3.2. Evaluate on Gait3D Dataset

On the Gait3D, GaitCSF also demonstrates significant performance advantages, as shown in Table 4. Compared to the silhouette-based method DANet, GaitCSF’s Rank-1 recognition rate improved by 13.15%; compared to the skeleton-based method SkeletonGait, it improved by 23.05%; compared to the multi-modal method HybridGait, it improved by 7.85%. In terms of mean average precision (mAP) and mean inverse negative penalty (mINP) metrics, GaitCSF also achieved excellent results of 52.82% and 33.94%, respectively, comprehensively surpassing the comparison methods. GaitCSF’s outstanding performance on the Gait3D validates its application potential in real commercial scenarios, especially its superior performance in handling complex conditions such as multi-viewpoint, partial occlusion, and irregular gait. The results thoroughly validate GaitCSF’s effectiveness in boosting gait recognition accuracy and robustness.

#### 4.3.3. Evaluate on SUSTech1K Dataset

In the evaluation on the SUSTech1K, the GaitCSF model fully demonstrates its comprehensive performance advantage in gait recognition tasks across diverse scenarios, as shown in Table 5. This dataset encompasses rich real-world scenario variations, including normal walking, item carrying, clothing changes, umbrella usage, partial occlusion, and low-light environments. Experimental results show that GaitCSF achieves a 73.24% overall accuracy in these comprehensive scenarios, strongly proving that the model possesses excellent environmental adaptability and can effectively address gait recognition challenges in various complex real-world scenarios.

### 4.4. Ablation Studies

To deeply analyze the effectiveness and contribution degree of each component of the GaitCSF model, we conducted a series of ablation experiments on the GREW. The experiments follow the same training configuration and evaluation protocol to ensure the comparability and consistency of results. Through these experiments, we systematically evaluated the individual contributions of CFS and SFL and their synergistic effects. To verify the effectiveness of each module design, we conducted a series of ablation control experiments based on the GaitBase benchmark framework on the Grew large-scale outdoor gait dataset. All ablation experiments strictly adopt the hyperparameter settings listed in Table 6, ensuring reliable comparability and consistency between experimental results.

#### 4.4.1. Evaluate CFS Module

CFS brings an 11.98% improvement in Rank-1 accuracy, proving the effectiveness of channel shuffling and group feature regulation mechanisms for feature optimization. This significant improvement mainly stems from CFS’s unique design philosophy: dividing feature channels into multiple subspaces for independent processing, then promoting communication and fusion between different semantic information through channel shuffling operations. This design is particularly suitable for processing multi-modal gait features, enhancing feature expressiveness and discriminative power while maintaining computational efficiency.

To deeply understand its working mechanism, we conducted detailed analysis of its key parameters and internal structure. CFS contains two main branches: the channel regulation branch and the spatial regulation branch. The channel branch captures channel-level statistical information through global average pooling, generating channel weight vectors; the spatial branch generates spatial sensitivity maps through group normalization, both dynamically adjusted through learnable parameters. This dual-dimensional feature regulation strategy can simultaneously optimize the importance of inter-channel and spatial positions, providing more refined feature representation.

CFS channel shuffling operations promote information exchange between different subgroups, enhancing feature expression diversity. Cross-group information exchange is important for constructing high-quality feature representations. Channel shuffling operations break the isolation between subgroups, allowing each output channel to contain information from different semantic groups, significantly enhancing the discriminative power of features.

Overall, the ablation experimental results of CFS fully prove its effectiveness and efficiency in gait feature optimization. Through channel grouping, dual-dimensional feature regulation, and channel shuffling operations, it can achieve adaptive enhancement of features with relatively low computational overhead, significantly improving the model’s recognition performance.

#### 4.4.2. Evaluate SFL Module

Experimental results show that the introduction of SFL improved Rank-1 recognition rate by 10.21%, validating the importance of frequency domain transformation in gait feature extraction. By mapping spatial domain features to the frequency domain through Fourier transform, SFL can effectively capture periodic patterns and global dependencies in gait sequences, which is crucial for gait recognition. The global receptive field advantage of frequency domain analysis allows the model to transcend the spatial limitations of local convolution operations, extracting more stable gait features, especially performing excellently when processing large-scale data in complex outdoor environments. SFL adopts a dual-path design, with one path processing features in the spatial domain and the other mapping features to the frequency domain for processing, finally fusing these complementary information through an adaptive weighting mechanism. This spatial-frequency cooperative processing strategy can simultaneously capture local detail features and global periodic patterns, providing more comprehensive gait representation.

The ablation experimental results fully prove the importance and effectiveness of frequency domain feature enhancement in gait recognition. By mapping gait features to the frequency domain for analysis, the model can capture more comprehensive gait patterns and long-range dependencies, significantly improving recognition performance, especially exhibiting excellent performance when dealing with complex environments and irregular gaits.

#### 4.4.3. Evaluate CFS and SFL Module

When CFS and SFL modules are used simultaneously, model performance reaches optimum, with Rank-1 accuracy improving by 15.36% compared to the baseline model. CFS optimizes feature representation through channel grouping and feature shuffling, while SFL extracts global periodic features through frequency domain transformation, both enhancing the model’s feature expression capability. SFL performs excellently in processing temporal information and long-range dependencies, while CFS is more efficient in optimizing the importance of channel and spatial dimensions of features, complementing each other and providing multi-dimensional feature enhancement; the integration of both modules enables the model to perform feature optimization simultaneously in frequency and spatial domains, constructing more comprehensive and robust gait representation, particularly suitable for recognition tasks in complex environments.

Through a series of ablation experiments, results show that GaitCSF performs excellently under various challenging conditions, with advantages particularly evident when processing complex scenarios such as viewpoint changes, clothing differences, and partial occlusion. This proves that the various components of GaitCSF can effectively work together when addressing different types of challenges, improving the overall robustness and adaptability of the model. It also validates the contribution and necessity of each component of GaitCSF, while providing valuable reference for the design and optimization of multi-modal gait recognition methods, offering new technical paths for solving complex scenario challenges in gait recognition, with important research value and application prospects.

#### 4.4.4. Ablation Study on Different Modalities

To evaluate the contribution of each modality in our multi-modal framework, we conducted ablation experiments comparing the performance of models trained with different modal configurations. As shown in Table 7, using only silhouette data achieved a Rank-1 accuracy of 67.83% on the GREW dataset, while using only heatmap data yielded 64.54%. However, when both modalities were combined in our GaitCSF framework, the performance significantly improved to 75.46%, demonstrating a relative gain of 7.63% and 10.92%, respectively. This substantial improvement confirms the complementary nature of these two modalities: silhouette data effectively captures the overall body shape and movement contours, while heatmap data provides precise structural information about key body joints and their dynamic relationships. Our multi-modal approach successfully leverages the strengths of both modalities while mitigating their individual limitations, leading to more comprehensive and robust gait representations across diverse real-world scenarios.

#### 4.4.5. Evaluate CFS Module

### 4.5. More Experiments

In this section, GaitCSF will undergo additional experiments. Beyond evaluating the aforementioned modules, we will assess the contribution of CFS and SFL components by conducting a comparative analysis between the baseline architecture (without these integrated modules) and the complete GaitCSF framework. This comparison will help quantify the performance enhancements attributable to these specialized components. Furthermore, we will conduct a comprehensive complexity analysis of GaitCSF to determine the effectiveness of CFS and SFL modules.

#### 4.5.1. Feature Visualization

**Figure 8:**
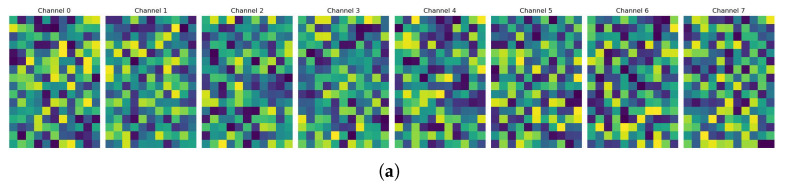

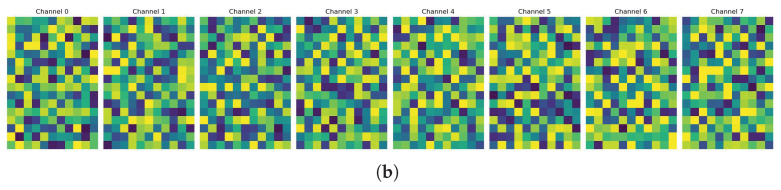
Feature maps of the baseline model of GaitCSF and the model integrated with CFS and SFL modules. (**a**) Baseline model. (**b**) GaitCSF integrated with CFS and SFL modules.

In the feature analysis section, we employ feature map visualization techniques to conduct an in-depth analysis of high-dimensional feature representations in the intermediate layers of the network. This methodology, based on intuitive representations of feature activation patterns, facilitates understanding of the network’s internal feature learning mechanisms. We extract feature tensors with dimensions (N, C, H, W) from the high-level layers of the network. To ensure the representativeness of visualization results, we select a single sample and sample 8 channels from its 256 feature channels for display analysis. In the preprocessing stage, we perform standardization transformations on each feature channel to eliminate scale differences between channels. Specifically, we calculate the mean μ and standard deviation σ of the feature map, and apply the Z-score normalization formula: $x^{\prime }=\frac{x-\mu }{\sigma }$. Subsequently, through linear transformation, we map the standardized feature values to the [0, 255] interval and perform data type conversion to obtain 8-bit integer representation. In terms of visualization presentation, as illustrated in Figure 8, we employ the perceptually uniform viridis colormap, which exhibits superior numerical-to-visual mapping characteristics. Feature activation intensity is encoded through color luminance, with high-activation regions rendered in bright tones and low-activation regions displayed in dark tones. Through this methodology, we ultimately present the feature maps of both the baseline model of GaitCSF and the model integrated with CFS and SFL modules.

As shown in Figure 8, we visualize the feature maps from both the baseline model (Figure 8a) and the GaitCSF model with integrated CFS and SFL modules (Figure 8b). These visualizations provide intuitive insights into how our proposed modules enhance feature representation capabilities.The feature maps from the baseline model (Figure 8a) show relatively uniform activation patterns with less distinct focus areas. The activation intensity is more evenly distributed across spatial locations, indicating that the baseline model extracts features without strong emphasis on specific discriminative regions. In contrast, the feature maps from the GaitCSF model (Figure 8b) demonstrate more pronounced activation patterns with higher contrast between highly activated regions (bright areas) and less activated regions (dark areas). This indicates that the CFS and SFL modules significantly enhance the model’s ability to focus on discriminative gait features. We observe more concentrated activation patterns in key body regions, suggesting the model’s improved attention to motion-relevant areas. The enhanced inter-channel variation demonstrates the effectiveness of the channel shuffle mechanism in promoting information exchange between different semantic groups. Additionally, stronger boundary definitions in the feature maps indicate the model’s improved capability to capture detailed spatial characteristics of gait patterns.

These visualization results confirm that the integration of CFS and SFL modules enables GaitCSF to learn more discriminative and focused feature representations, which directly contributes to its superior recognition performance compared to the baseline model.

#### 4.5.2. Complexity Analysis

Compared to the baseline model GaitBase with 19.29030 M parameters, GaitCSF has 39.25140 M parameters, representing an increase of 19.9611 M parameters. This significant growth primarily stems from the multi-modal three-branch parallel architecture design adopted by GaitCSF. GaitCSF comprises three independent ResNet-Like backbone networks that process silhouette features, heatmap features, and their fusion features, respectively, while GaitBase employs only a single-branch architecture. Despite the substantial increase in parameters, this design yields remarkable performance improvements: accuracy is significantly enhanced on both GREW and SUSTech1k datasets. The three independent feature extraction branches ensure that each modality’s features can be fully developed within their specific feature spaces, avoiding information loss caused by simple fusion. While this design increases the parameter count, it significantly enhances feature representation capability.

Through the introduction of CFS and SFL modules, GaitCSF significantly enhances gait feature extraction and discrimination capabilities while maintaining relatively lightweight model parameters. Particularly when handling complex environmental variations and different datasets, the model demonstrates robust performance. Specifically, the CFS module achieves substantial improvement in feature representation capability through channel grouping and feature shuffling strategies. This module realizes cross-channel information interaction and feature enhancement with minimal parameter overhead through fine-grained feature regulation mechanisms. The SFL module, by introducing frequency domain transformation and dual-path processing architecture, effectively captures periodic patterns and global dependencies in gait sequences with limited additional parameters, significantly improving the model’s capability to model long-range temporal information.

This performance improvement not only results from the moderate increase in model complexity but, more importantly, from GaitCSF’s optimized feature learning capability, enabling the model to better capture critical dynamic information in gait, especially when confronting complex scenarios such as viewpoint variations, clothing differences, and occlusions. Experimental results demonstrate that GaitCSF, through its carefully designed parallel architecture and efficient feature enhancement modules, achieves an excellent balance in parameter utilization efficiency, providing an effective solution for gait recognition tasks in complex environments.

## 5. Conclusions

This paper proposes a multi-modal gait recognition network based on channel shuffle regulation and spatial-frequency joint learning (GaitCSF), effectively addressing the poor performance of traditional single-modal gait recognition methods in complex scenarios by fusing silhouette data and heatmap data. The core innovations of GaitCSF lie in introducing the channel feature selection (CFS) module based on channel shuffling and the spatial-frequency joint learning (SFL) module, with the former achieving adaptive enhancement of features through channel grouping and feature shuffling strategies, and the latter effectively capturing periodic patterns and global dependencies in gait sequences through frequency domain transformation. The model adopts a three-branch parallel feature extraction architecture, fully leveraging the complementary advantages of silhouette features and heatmap features.

Testing conducted across the three benchmark datasets GREW, Gait3D, and SUSTech1k reveals that GaitCSF exceeds the performance of current methodologies on every evaluation criterion. On the GREW large-scale outdoor dataset, GaitCSF achieves a Rank-1 recognition rate of 75.46%; on the Gait3D real supermarket scene dataset, it achieves a Rank-1 recognition rate of 66.15% and mAP of 52.82%; on the SUSTech1k multi-attribute test set, it achieves an overall accuracy of 73.24%. The findings comprehensively confirm GaitCSF’s innovation and efficacy within gait recognition research.

In conclusion, this paper provides new technical ideas and solutions for the field of gait recognition, laying the foundation for identity recognition applications in complex scenarios. GaitCSF not only surpasses existing methods in performance but also provides new research perspectives for gait feature extraction and multi-modal fusion, with important theoretical value and application prospects. In the future, we will continue to deepen related research, further enhancing the performance and robustness of gait recognition systems in practical applications, promoting the widespread application of this technology in fields such as smart cities, public security, and identity verification.

## 6. Discussion

Compared to existing gait recognition methods, GaitCSF has several significant advantages: it performs more stably in complex scenarios, effectively addressing practical challenges such as viewpoint changes, clothing differences, partial occlusion, and lighting changes; it utilizes multi-modal information more efficiently, achieving deeper-level modal fusion through unique frequency domain enhancement and feature regulation mechanisms. The experimental results across GREW, Gait3D, and SUSTech1k datasets confirm this robustness, with performance improvements of 7.58% over silhouette-based GaitRGA, 21.86% over skeleton-based GPGait, and 11.46% over multi-modal GaitSTR on the GREW dataset. Despite GaitCSF’s significant progress, there are still some directions worthy of further exploration: first, determining how to more effectively handle gait recognition under extreme environmental conditions (such as severe occlusion, dramatic viewpoint changes) remains a challenge; second, the current model mainly focuses on two modalities—silhouette and heatmap—future work could explore introducing more modal data to further enhance the model’s expressive ability; third, determining how to design more lightweight model architectures, reducing computational complexity and memory usage to adapt to resource-constrained practical application scenarios; this will also be our future research focus.

Although this research has made significant progress, there are still some directions worthy of further exploration:

- Determining how to enhance model robustness under extreme lighting and viewpoint changes;
- Reducing computational complexity while maintaining high recognition accuracy;
- Exploring more efficient temporal modeling strategies to better capture dynamic information in gait sequences. These directions will be the focus of our future research.

In conclusion, this paper provides new technical ideas and solutions for the field of gait recognition through channel shuffle regulation and spatial-frequency joint learning, laying the foundation for identity recognition applications in complex scenarios. In the future, we will continue in-depth research to further enhance the accuracy and robustness of gait recognition systems in practical applications.

## Figures and Tables

**Table 1:** Number of IDs and Seqs in the Grew, Gait3D, and SUSTech1k datasets.

	Train Set		Test Set	
Datasets	#ID	#Seq	#ID	#Seq
Grew	20,000	102,887	6000	24,000
Gait3D	3000	18,940	1000	6369
SUSTech1k	200	5988	850	19,228

**Table 2:** Hyperparameters used for all experiments.

Dataset	BatchSize	Steps	Mutistep Scheduler
Grew	(32, 2)	180,000	(80 K, 120 K, 150 K)
Gait3D	(32, 2)	60,000	(20 K, 40 K, 50 K)
SUSTech1K	(8, 16)	50,000	(20 K, 40 K)

**Table 3:** Comparison of GaitCSF with existing SOTA methods on the Grew dataset.

Input	Method	Venue	Rank-1	Rank-5	Rank-10	Rank-20
Silhouette-based	GEINet [43]	ICB2016	6.82	13.42	16.97	21.01
	GaitSet [39]	AAAI2019	46.30	63.60	70.30	76.90
	GaitPart [15]	CVPR2020	44.00	60.70	67.40	73.50
	GaitGL [18]	ICCV2021	51.40	67.50	72.80	77.30
	GaitBase [41]	CVPR2023	60.10	75.50	80.40	84.10
	GaitSSB [44]	CVPR2023	61.70	-	-	-
	QAGait [32]	AAAI2024	59.10	74.00	79.20	83.20
	GaitRGA [45]	MDPI2025	67.88	82.14	86.31	89.35
Skeleton-based	GaitGraph2 [46]	CVPRW2022	33.50	49.40	56.20	61.90
	GaitTR [47]	ES2023	54.50	-	-	-
	GPGait [33]	ICCV2023	53.60	-	-	-
Multi-model	GaitRef [34]	IJCB2023	53.00	67.90	73.00	77.50
	TransGait [48]	APPL INTELL2023	56.27	72.72	78.12	82.51
	MSAFF [26]	IEEE2024	57.40	72.99	78.27	82.88
	GaitSTR [35]	CVPR2024	64.00	78.50	83.20	86.30
	GaitCSF	Ours	75.46	87.15	91.09	93.11

**Table 4:** Performance of GaitCSF on the Gait3D dataset, evaluated in terms of Rank-1, Rank-5, mAP, and mINP.

Input	Method	Venue	Rank-1	Rank-5	Rank-10	Rank-20
Silhouette-based	GEINet [43]	ICB2016	5.40	14.20	5.06	3.14
	CSTL [40]	ICCV2021	11.70	19.20	5.59	2.59
	GaitPart [15]	CVPR2020	28.20	47.60	21.60	12.40
	GaitGL [18]	ICCV2021	29.70	48.50	22.30	13.60
	GLN [49]	ECCV2020	31.40	52.90	24.74	13.58
	GaitSet [39]	AAAI2019	36.70	58.30	30.00	17.30
	DANet [50]	CVPR2023	48.00	69.70	-	-
Skeleton-based	GaitGraph2 [46]	CVPRW2022	11.10	24.00	-	-
	GaitTR [47]	ES2023	6.60	-	-	-
	GPGait [33]	ICCV2023	22.50	-	-	-
	SkeletonGait [22]	CVPR2024	38.10	56.70	28.90	16.10
Multi-model	SMPLGait [30]	CVPR2022	46.30	64.50	37.20	22.20
	MSAFF [26]	IEEE2024	48.10	66.60	38.45	23.49
	GaitRef [34]	IJCB2023	49.00	49.30	40.70	25.30
	HybridGait [24]	AAAI2024	53.30	72.00	43.30	26.70
	GaitCSF	Ours	61.15	77.51	52.82	33.94

**Table 5:** Rank-1 accuracy comparison on SUSTech1K with other leading models.

Method	Venue	Normal	Bag	Clothing	Carrying	Umbrella	Uniform	Occusion	Night	Overall
GaitGraph2 [46]	CVPRW2022	22.20	18.20	6.80	18.60	13.40	19.20	27.30	16.40	18.60
GaitTR [47]	ES2023	33.30	31.50	21.00	30.40	22.70	34.60	44.90	23.50	30.80
SkeletonGait [22]	CVPR2024	55.00	51.00	24.70	49.90	42.30	52.00	62.80	43.90	50.10
GaitPart [15]	CVPR2019	62.20	62.80	33.10	59.85	58.21	58.74	60.82	22.17	59.72
GaitRGA [45]	MDPI2025	65.70	62.43	30.01	59.50	57.20	54.80	57.20	21.70	59.20
GaitGL [18]	ICCV2021	67.10	66.20	35.90	63.30	61.60	58.10	66.60	17.90	63.10
GaitSet [39]	AAAI2019	69.10	68.20	37.40	65.00	63.10	61.00	67.20	23.00	65.00
BiFusion [51]	MTA2023	69.80	62.30	45.40	60.90	54.30	63.50	77.80	33.70	62.10
GaitCSF	Ours	73.24	73.22	45.54	71.61	66.53	67.73	77.84	41.31	70.75

**Table 6:** Results of ablation experiments for each module of GaitCSF (%).

Method	CFS	SFL	Rank-1	Rank-5	Rank-10	Rank-20
Baseline			60.1	75.50	80.40	84.10
	✓		72.08	85.12	88.78	91.42
		✓	70.31	83.94	87.98	90.89
	✓	✓	75.46	87.15	91.09	93.11

**Table 7:** Contributions of different modalities to GaitCSF (%).

Method	Silhouette	Heatmaps	Rank-1	Rank-5	Rank-10	Rank-20
GaitCSF	✓		67.83	83.89	87.12	90.91
		✓	64.54	80.45	84.77	88.76
	✓	✓	75.46	87.15	91.09	93.11

## Data Availability

Due to the licenses for the GREW, Gait3D, and SUSTech1k raw datasets, we do not provide data on method use here. Please apply yourself if needed: GREW: https://github.com/XiandaGuo/GREW-Benchmark/blob/main/docs/0.download_datasets.md (accessed on 16 July 2024); Gait3D: https://gait3d.github.io (accessed on 25 August 2024); SUSTech1K: https://lidargait.github.io (accessed on 12 June 2024).

## Abbreviations

List of symbols and terminology.

**Symbol/Term**	**Description**
GaitCSF	Multi-modal gait recognition network based on channel shuffle regulation and
	spatial-frequency joint learning
CFS	Channel shuffle-based feature selective regulation module
SFL	Spatial-frequency joint learning module
TP	Temporal pooling
HPP	Horizontal pyramid pooling
FC	Fully connected layer
BNNeck	Batch normalization neck network
Silhouette	Binary human silhouette data
Heatmap	Skeleton heatmap data
*N*	Batch size
*C*	Feature channel dimension
*S*	Sequence length
*H*	Feature map height
*W*	Feature map width
*e*	Input feature maps
*E*	Output feature vectors
*K*	Input feature for CFS module
$K_{x}$	Channel subspace features
$K_{x}^{sp}$	Features after spatial processing
$K_{x}^{ch}$	Features after channel processing
*X*	Input feature for SFL module
$X_{freq}$	Features after frequency domain transformation
$X_{spatial}$	Features after spatial domain processing
*Y*	Module output features
⊕	Feature concatenation operation
σ	Sigmoid activation function
FFT2D	2D fast Fourier transform
GN	Group normalization
GAP	Global average pooling
BN	Batch normalization
ReLU	Rectified linear unit activation function
Rank-*k*	Rank-*k* recognition accuracy
mAP	mean average precision
mINP	mean inverse negative penalty
